# Identification and Characterization of a Plant Endophytic Fungus *Paraphaosphaeria* sp. JRF11 and Its Growth-Promoting Effects

**DOI:** 10.3390/jof10020120

**Published:** 2024-01-31

**Authors:** Jie Shan, Fangren Peng, Jinping Yu, Qi Li

**Affiliations:** 1 Jiangsu Key Laboratory for the Research and Utilization of Plant Resources, Institute of Botany, Jiangsu Province and Chinese Academy of Sciences (Nanjing Botanical Garden Mem. Sun Yat-Sen), Nanjing 210014, China; m17354973215@163.com (J.S.); yujinping@cnbg.net (J.Y.); 2 College of Forestry, Nanjing Forestry University, Nanjing 210037, China; frpeng@njfu.edu.cn

**Keywords:** endophytic fungi, *Paraphaosphaeria*, growth-promoting

## Abstract

Endophytic fungi establish mutualistic relationships with host plants and can promote the growth and development of plants. In this study, the endophytic fungus JRF11 was isolated from *Carya illinoinensis*. Sequence analysis of the internal transcribed spacer (ITS) region and *18S rRNA* gene combined with colonial and conidial morphology identified JRF11 as a *Paraphaosphaeria* strain. Plant–fungus interaction assays revealed that JRF11 showed significant growth-promoting effects on plants. In particular, JRF11 significantly increased the root biomass and soluble sugar content of plants. Furthermore, transcriptome analysis demonstrated that JRF11 treatment reprogrammed a variety of genes involved in plant mitogen-activated protein kinase (MAPK) signaling and starch and sucrose metabolism pathways through Kyoto Encyclopedia of Genes and Genomes (KEGG) enrichment analysis. Our research indicates that beneficial endophytic fungi are able to interact with plants and exhibit outstanding plant growth-promoting activities.

## 1. Introduction

There are many kinds of endophytic fungi in multiple plants, including algae, gymnosperms, and angiosperms. Over the long course of evolution, endophytic fungi and plants have gradually formed symbiotic relationships instead of allowing them to become isolated entities [1]. The interactions between these endophytic fungi and plants beneficially impact plant survival, fitness, biodiversity, and ecosystemic function [2]. Clavicipitaceous (usually associated with grasses) and nonclavicipitaceous (not found in grasses) are usually considered to be two major groups of endophytic fungi [3]. Among them, *Trichoderma* and *Aspergillus* spp. have been frequently reported as beneficial fungi in recent studies [4,5]. Endophytic fungi can effectively promote plant growth and improve plant disease resistance. Sareh Hatamzadeh et al. found that many strains of endophytic fungi, such as *Preussia Africana*, *Fusarium avenacearum*, and *Alternaria embellisia*, significantly improved the germination of maize hybrid-cultivar seeds [6]. Furthermore, Huang et al. discovered that 35 endophytic fungi isolated from cucurbit plants showed obvious growth-promoting effects and had excellent potential for plant disease control [7]. Therefore, isolation and utilization of endophytic fungi strains with appropriate growth-promoting capacities on crops may help reduce the use of fertilizer and fungicide in agro-ecosystems [8].

After symbiosis with host plants, endophytic fungi not only promote the growth of host plants but also reduce the negative influence of the harsh environment on host plants. Hiran Kanti Santra et al. found that the endophytic fungus *Colletotrichum alatae* LCS1, which was isolated from *Lycopodium clavatum*, interacted with plants and led to remarkable drought resistance in plants [9]. Rafał Ważny et al. discovered that plants inoculated with the endophytic fungi *Mucor* sp. or *Trichoderma asperellum* could adapt to unfavorable environments [10]. Similarly, Adeela Naureen isolated the root-endophytic fungus CJAN1179 from *Cymbopogon jwarancusa* and observed that CJAN1179 significantly enhanced the number of *Arabidopsis thaliana* (Col-0) lateral roots (increased 3.1-fold) [11]. Previous studies indicated that inoculation with dark septate endophytic (DSE) fungi promoted the growth of tomato plants. In particular, DSE fungi increased the content of nitrogen and the efficiency of fertilizer-K recovery [12]. In addition, Chithra S et al. studied the endophytic fungus *Phomopsis* sp. isolated from *Piper nigrum* for its colonization ability on *Oryza sativa*. Interestingly, the plants treated with this fungus displayed a significant growth enhancement [13]. Finally, the effect of endophytic fungi on plant growth is far from well-studied, and certain kinds of endophytic fungi are waiting for us to explore.

*Paraphaosphaeria* is a new genus that has been discovered in recent years [14]. In most studies, endophytic fungi belonging to the genus *Paraphaosphaeria* were reported to improve disease resistance in plants. This is mainly because the interaction between endophytic fungi and host plants could induce the production of antifungal metabolites against pathogens [15]. Su et al. isolated the fungus *Paraphaeosphaeria* sp. YXG-18 from the medicinal plant *Ginkgo biloba* and found that YXG-18 exhibited significant antifungal activities against the phytopathogen *Alternaria* spp. [16]. Li et al. observed that endophytic fungi produced plenty of biologically active secondary metabolites, which could inhibit some pathogenic bacteria, including S. *aureus*, C. *difficile*, and *P. aeruginosa* PAO1 [17]. Whether *Paraphaeosphaeria* endophytic fungi can promote plant growth has not been studied yet.

*Carya illinoinensis* is an important economic forest species, and its fruit has high nutritional values [18]. To date, endophytic fungi in *C. illinoinensis* and their function have not been reported. In this study, we isolated 14 endophytic fungi from *C. illinoinensis* and identified them by sequence analysis of the internal transcribed spacer (ITS) region and *18S rRNA* gene combined with colonial and conidial morphology. Among them, JRF11 significantly increased plant biomass and improved lateral root growth. Meanwhile, we found that the soluble sugar of tomato seedlings also notably increased under JRF11 treatment. Through RNA-seq analysis, we found that Gene ontology (GO) terms were mainly concentrated in oxygen-related pathways, and Kyoto Encyclopedia of Genes and Genomes (KEGG) major enriched pathways were “mitogen-activated protein kinase (MAPK) signaling pathway-plant” and “starch and sucrose metabolism”. Our results demonstrate that symbiotic interactions between JRF11 and plants can effectively promote plant growth, suggesting that JRF11 is capable of acting as a good potential fertilizer for agricultural development.

## 2. Materials and Methods

### 2.1. Isolation of Endophytic Fungi

Roots and stems of *Carya illinoinensis* were prepared and rinsed with water, then cut evenly and placed on filter paper. Firstly, the washed samples were placed in a 10 mL centrifuge tube and repeatedly shaken with sterile water. Secondly, the samples were cleaned and sterilized using a 75% ethanol solution, and the supernatant was removed following natural sedimentation [19,20,21,22,23]. Then, samples were rinsed three times with sterile water to eliminate the ethanol. Finally, the 10% sodium hypochlorite solution was added, and the samples were repeatedly shaken and cleaned. Following sterilization, the supernatant was removed naturally, and the sterile water was repeatedly added to remove sodium hypochlorite by shaking. This process was performed three times.

After sterilization on an ultra-clean bench, the samples were sliced with a little knife and placed on a potato dextrose agar (PDA) plate at 27 °C in the dark until the mycelium appeared. The mycelium was transferred to new plates, isolated, purified to acquire pure strains, and stored in the dark [24,25].

### 2.2. Molecular Identification of Isolated Fungi

First, three mycelium plugs (5 mm diameter) were placed into a 2 mL tube with 500 μL of 0.01 M phosphate buffer (pH 7.2–7.4), and the mixture was shaken and mixed well. Second, the appropriate amount of sterilized glass beads with a diameter of 0.5 mm and 0.1 mm were added to the tube, and then the mixture was ground with an oscillator at a speed of 6.5 m/s for 1 min. Finally, the samples were centrifuged at 12,000 rpm for 2 min, and the supernatant was taken into 1.5 mL sterilized tubes for testing.

To identify endophytic fungi, polymerase chain reaction (PCR) was performed to amplify the internal transcribed spacer (ITS) region and *18S rRNA* gene of the fungal isolates using universal primers (ITS1, 5′-TCCGTAGGTGAACCTGCGG-3′ and ITS4, 5′-TCCTCCGCTTATTGATATGC-3′, or NS1, 5′-GTAGTCATATGCTTGTCTC-3′ and NS6, 5′-GCATCACAGACCTGTTATTGCCTC-3′). The amplified PCR products were checked on a 1% agarose gel and then sent to Sangon Biotech (Shanghai, China) Co., Ltd. for sequencing. All obtained sequences were analyzed using a Basic Local Alignment Search Tool (BLAST) search in the database of the National Center of Biotechnology Information (NCBI) to find the closest matches.

To conduct the phylogenetic analysis, the partial ITS region and *18S rRNA* gene sequences of 10–15 species with high similarity were selected and aligned by MEGA version 7.0 software using the ClustalW method. Phylogenetic trees were constructed using the maximum likelihood method with 100 bootstrap replicates.

### 2.3. Co-Culturation of Seedlings with Endophytic Fungi

#### 2.3.1. Sterilization of Seeds

For surface sterilization, a specified quantity of *Arabidopsis thaliana*/tomato seeds was placed in 2 mL centrifuge tubes. Then, the sterilization of seeds was conducted according to the methods described above (Section 2.1). Briefly, the seeds were successively sterilized with a 75% ethanol solution and a 10% sodium hypochlorite solution and rinsed in sterile water. Finally, the seeds were resuspended in 1 mL of sterile water.

#### 2.3.2. Co-Culturation

The sterilized *Arabidopsis thaliana*/tomato (cultivar: Da Hong tomato) seeds were spread evenly on 1/2MS solid medium, and sterile water was sucked out of the medium. They were dried at the ultra-clean bench and sealed with sealing film, transferred to the artificial climate chamber, and cultured vertically at 22 °C for 16 h/8 h until germination [26,27].

Five millimeters of PDA plugs and 5 mm of mycelium plugs were placed on the left and right sides of the 1/2MS solid medium, 2.5 cm away from the edge of the Petri dishes. After the seeds were germinated, the seeds with the same growth vigor were selected and placed on the Petri dishes. Three seedlings on each side were sealed with a sealing membrane and transferred to the artificial climate chamber. They were cultured vertically at 22 °C for 16 h/8 h for 7–10 days until there were visible differences.

### 2.4. Potted Growth-Promoting Test of Endophytic Fungi

Tomato (cultivar: Da Hong tomato) seedlings were prepared in advance, and after one week, seedlings of uniform size were selected and transplanted into pots (10 cm × 10 cm) containing nutrient soil. One seedling was transplanted in one pot, and five pots were placed in each tray as five replicates. Each treatment set up a total of two trays of 10 replicates. Freshly cultured endophytic fungi (3–5 mycelium plugs) were obtained with a 5 mm puncher and added into 200 mL of potato dextrose broth (PDB) medium, cultured at 25 °C and 160 rpm for 7 days. Then, the fermentation broth was collected. First, the fermentation broth was filtered with gauze, and then the filtrate was diluted with sterile distilled water to 400 mL as the stock solution. Four treatments were set up for tomatoes: sterile distilled water, a 10% stock solution, a 20% stock solution, and a 50% stock solution, respectively. During treatment, 40 mL of solution was poured into each pot along the main root of the plant, and 200 mL of solution was required for each tray.

A week after transplanting, the roots of tomato seedlings were irrigated with stock solution and then treated the second time after 10 days, repeated three times. During the period, plant height, leaf number, SPAD, and other data were measured. The plant materials were collected until the growth difference between the treatments was visible, and the data on fresh weight, root length, dry weight, and nutrient element content were measured [28].

### 2.5. Determination of Total Nitrogen, Phosphorus, and Potassium

First, the samples were dried in a drying oven at 80 °C–90 °C for 15–30 min, then they were cooled to 60 °C–70 °C after the water was drained. Plant materials were pulverized, screened with a 40-mesh sieve, and combined together. The 0.005–0.2 g of samples, exact to 0.0001 g, were placed into a 300 mL desiccation pipe (do not stick the sample to the bottle’s neck). Then, the sample was wet with a little water and 5 mL of sulfuric acid, and 2 g of accelerator was added.

A small funnel with a curved neck was placed at the mouth of the tube, and the funnel was boiled at 250 °C in the retort furnace (the temperature was stable for about 30 min.). When the H_2_SO_4_ was decomposed and a lot of white smoke was emitted, the temperature was raised to 400 °C. The solution was removed when it was a uniform brownish-black color (the time was about 3 h). Finally, the solution was measured for the content of nitrogen (N), phosphorus (P), and potassium (K) when it was cooled. Then, a blank sample was also measured.

South China Agricultural University. Kelvin-distillation titration was used for total nitrogen (TN) determination; the sodium hydroxide melting-molybdenum antimony anti-colorimetric method was used for total phosphorus (TP) determination; and sodium hydroxide melting-flame atomic absorption spectrophotometry was used for total potassium (TK) determination [29,30].

### 2.6. Determination of Total Soluble Sugar

#### 2.6.1. Production of Standard Curve

To begin, 0, 0.2, 0.4, 0.8, 1.0, 1.5, and 2.0 mL of a 100 μg/mL sucrose solution were added to several 20 mL graduated test tubes, and the total volume was made up to 2 mL with water. Then, 0.5 mL of ethanol solution and 5 mL of sulfuric acid were successively added into the test tubes and thoroughly vibrated. After that, the test tubes were immediately immersed in a boiling water bath. Each tube was accurately kept warm for 1 min before being removed and naturally cooled to a normal temperature, with a blank sample serving as a control. The absorbance was measured at 630 nm, and the standard curve was plotted with absorbance as the horizontal coordinate and sugar content as the vertical coordinate, and the standard linear equation was founded.

#### 2.6.2. Extraction of Soluble Sugar in Samples

A 15 mL centrifuge tube with 0.02–0.20 g of sample was taken, 10 mL of distilled water was added to it, and the sample was placed in a boiling water bath for 30 min. The solution was cooled and centrifuged, and the supernatant was transferred to a 25 mL volumetric flask, and 10 mL of distilled water was added to it. The centrifuge tubes and other residue were rinsed several times, and then the volume was fixed to the scale. Finally, the samples were filtered before determination.

#### 2.6.3. Colorimetric Determination

One milliliter of sample diluent was added to a 20 mL graduated test tube with 1 mL of water. Then, 0.5 mL of ethanol solution acetate reagent and 5 mL of sulfuric acid were successively added to the test tube. It would be colored and used to determine absorbance. Finally, the amount of sugar (μg) was determined from the standard linear equation, and the sugar content in the test samples was calculated.

### 2.7. Determination of Soluble Protein Content

First, 0, 0.2, 0.4, 0.6, 0.8, and 1.0 mL of a 100 μg/mL standard protein solution were pipetted respectively, and distilled water was added to make the total volume to 1 mL. Then, 5 mL of Coomassie brilliant blue solution was pipetted and set for 20 min. A blank sample was used as a control. The absorbance was measured at a wavelength of 595 nm. The standard curve regards absorbance as the abscissa and the content of protein as the ordinate. Finally, the standard linear equation was obtained.

The 0.02–0.30 g samples were taken into mortar, 2 mL of distilled water was added in for grinding, and the homogenate was transferred to a centrifugal tube. Then, the mortar was rinsed with 6 mL of water. The solution was collected in the centrifugal tube, centrifuged at 4000 rpm for 20 min, and the sediment was thrown away. The supernatant was transferred to a 10 mL volumetric flask, and the volume was fixed with distilled water. A 0.5 mL sample was pipetted, and its absorbance was measured.

### 2.8. Transcriptome Analysis

JRF11- and control-treated samples were used for total RNA extraction and library construction. Total RNA was isolated, and library construction and sequencing were conducted by the Majorbio Bio-Pharm Technology Co., Ltd. (Shanghai, China). The quality of the total RNA was assessed using the Agilent Bioanalyser 2100 (Agilent Technologies, Santa Clara, CA, USA) prior to subsequent experiments. The BGISEQ-500 platform was used for RNA sequencing and generating raw data [31,32]. The raw reads of the transcriptome data were filtered with SOAPnuke 2.0 software to remove unsatisfactory reads with low quality, joint contamination, and excessive unknown bases.

The level of transcription of genes was calculated by FPKM (fragments per kilobase per million fragments) to quantify gene expression, and DESeq2 (v1.0) software was used to screen differentially expressed genes (DEGs). The screening criteria were |log2foldchange| ≥ 1 and *p* < 0.05. Based on the criteria (|fold change, FC| ≥ 2 and Q-value ≤ 0.001), DEGs were defined. The obtained DEGs were annotated into the Gene Ontology (GO) and Kyoto Encyclopedia of Genes and Genomes (KEGG) databases to obtain functional annotation and metabolic pathway enrichment analysis.

### 2.9. Statistical Analysis

All data were tested for homogeneity before two-way ANOVA (testing the effects of co-culturation of seedlings and endophytic fungi and whole plant, root biomass, and physiology) and one-way ANOVA (Tukey’s HSD test was used to detect significant differences in each parameter, *p* < 0.05) with IBM SPSS Statistics 27 software. Origin 2022 software was used to create figures.

## 3. Results

### 3.1. Isolation and Identification of Endophytic Fungi

**Figure 1:**
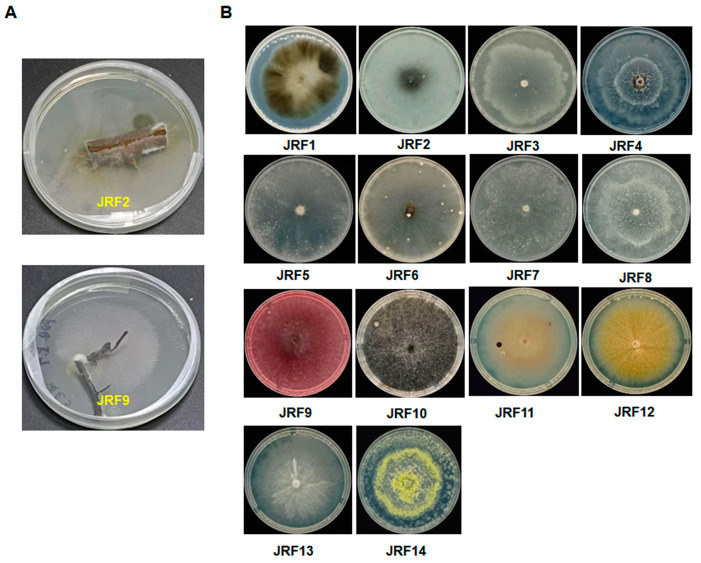
Fourteen strains of endophytic fungi isolated from *Carya illinoinensis*. Example for the isolation of endophytic fungi (**A**). Fourteen strains of endophytic fungi on PDA (**B**).

In this study, we obtained 20 purified strains of endophytic fungi at the initial stage; their morphological characteristics and phenotype appearances on potato dextrose agar (PDA) culture medium were observed and recorded. Based on this, 14 strains showing different morphological features were selected for further analysis. As shown in Figure 1 and Table 1, 14 strains of endophytic fungi were successfully isolated from different tissues of *Carya illinoinensis*, which included the stem and root. Among these endophytic fungi strains, six strains (42.9%) were isolated from roots, and eight strains (57.1%) were isolated from stems (Table 1). They were coded by serial numbers, namely, from JRF1 to JRF14. This result indicated that there were multiple endophytic fungi in the stem and root of the pecan.

We conducted the molecular biology identification of these fungal isolates via sequence analysis of the ITS (internal transcribed spacer) region. The ITS sequences of each isolate were generated using PCR amplification and submitted to the NCBI database to obtain the closest matches (Table 1). Among them, 14 strains were classified into nine genera, including *Aureobasidium*, *Botryosphaeria*, *Diaporthe*, *Fusearium*, *Mycoleptodiscus*, *Paraphaeosphaeria*, *Penicillium*, *Pseudthielavia,* and *Talaromyces* (Table 1). Moreover, the closest speices, sequence identity, accession numbers, and description of colonies of the identified endophytic fungal isolates were systematically cataloged and shown in Table 1.

### 3.2. Screening of Plant Growth-Promoting Endophytic Fungi

**Figure 2:**
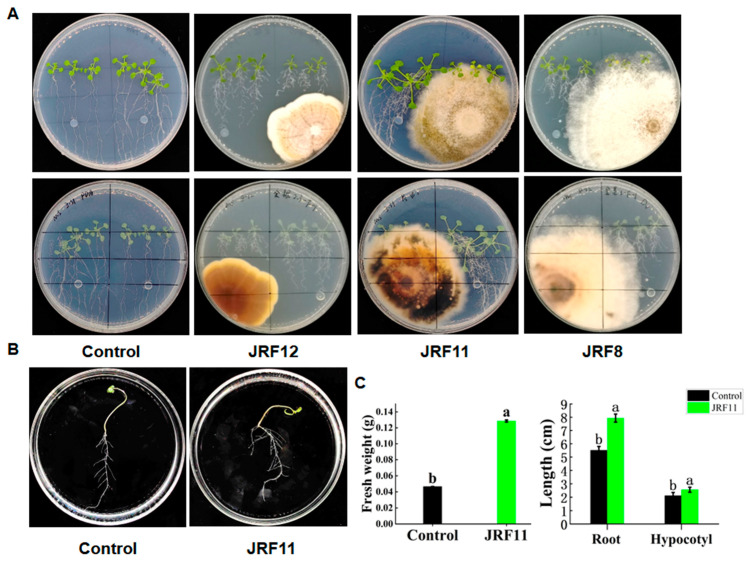
Growth-promoting tests of isolated fungi. Morphological characteristics of *Arabidopsis* seedlings inoculated with different fungi on 1/2MS medium (**A**). Pictures in the first and second rows showed the front and back views, respectively. Morphological characteristics of tomato seedlings inoculated with JRF11 (**B**). Fresh weight, root length, and hypocotyl length of tomato seedlings inoculated with JRF11 (**C**). The data are the means ± standard deviation of five replicates. Different letters indicate significant differences between all treatments, according to Duncan’s multiple-range test (*p* < 0.05).

To test whether these endophytic fungi can promote plant growth, we conducted plant–fungus interaction assays using *Arabidopsis* and all these fungi except for JRF2, JRF5, JRF7, and JRF9, which might be harmful to plants like phytopathogens in previous studies [33]. Then, inoculations of *Arabidopsis* seedlings with endophytic fungus were placed on 1/2 MS solid medium. After waiting for a period of time, these endophytic fungi displayed different effects on the growth of *Arabidopsis*. Overall, the JRF11-inoculated plants were larger and grew taller than the uninoculated controls, and they had more developed roots and green leaves (Figure 2A). Similar results were observed on JRF1-inoculated plants. However, the morphological traits of *Arabidopsis* inoculated with JRF4, JRF12, and JRF13 were comparable to those of the uninoculated control (Figure 2A and Table 1). Inoculation with JRF3, JRF6, JRF8, JRF10, and JRF14 inhibited the growth of *Arabidopsis*. Considering the growth-promoting effect of JRF11 was outstanding, we chose JRF11 for the next step.

### 3.3. Cultivation of Paraphaosphaeria sp. JRF11 and Its Plant Growth-Promoting Effects

Based on the above results, we also inoculated tomato seedlings with JRF11 on 1/2MS solid medium. Results showed that inoculation of JRF11 could significantly increase the growth of tomato seedlings on 1/2MS solid medium (Figure 2B). Compared with the control, the fresh weight of JRF11-inoculated plants significantly increased, and the root and hypocotyl length of JRF11-inoculated plants increased by 44.1% and 20.8% (Figure 2B,C). These data suggested that inoculation with JRF11 could significantly promote the growth of *Arabidopsis* as well as tomato plants.

In the preparatory test, JRF11 grew slowly on the MMN (Modified Melin-Norkrans) medium, which is commonly used. In order to find the most suitable medium for JRF11, we selected several basal mediums to screen, such as PDA, Yeast Extract Peptone Dextrose Medium, MMN Medium, Corn Meal Medium, Czapek Dox Agar Medium, Malt Extract Agar, and Sabouraud Dextrose Agar Medium [25]. Compared with others, the growth rate of JRF11 on PDA was 5.50 mm/d, and the sporulation quantity of JRF11 on Corn Meal Medium was more than 10/μL. And the growth rate of JRF11 on Corn Meal Medium was 5.00 mm/d. Based on this, we chose Corn Meal Medium for the culturation of JRF11 to observe its sporulation (Table 2).

### 3.4. Colonization of Plant Roots by Paraphaosphaeria sp. JRF11

**Figure 3:**
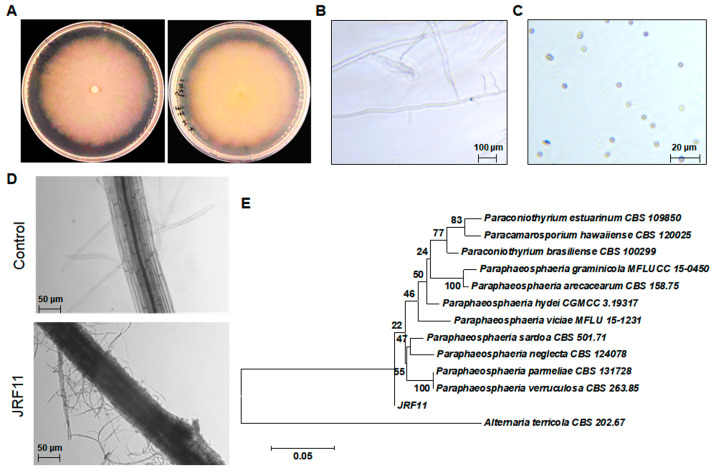
Morphological observation and identification of *Paraphaosphaeria* sp. JRF11. Visual observations of JRF11 (**A**). Left, front view; right, back view. Microscopic view of mycelium (**B**) and spores (**C**) of JRF11. Colonization of JRF11 on tomato seedlings (**D**). Phylogenetic tree based on ITS sequences (**E**). The value on each branch is the percentage of bootstrap replications supporting the branch. *Alternaria terricola* CBS 202.67 was used as an outgroup. Scale bar, 0.05 nucleotide substitutions per site.

Based on the above results, we conducted the morphological observation of JRF11 on Corn Meal Medium. The mycelium of JRF11 was rich, dense, and white on Corn Meal Medium, and the surface was covered by pure white aerial mycelium (Figure 3A). The reverse side was concolorous. JRF11 distributed multiple branches on the main stalk, and the apex of the hypha is solitary (Figure 3B). The conidia of JRF11 were one-celled, smooth in outline, 3.4 to 5.2 μm × 2.7 to 3.5 μm in size, and their shapes were spherical, ellipsoidal, or obovoid, and their colors were light yellow-brown, olivaceous-brown or translucent (Figure 3C), which were consistent with the observations in recent studies [34]. After that, we used JRF11 to inoculate tomato plants, and observed them after 10 d. Through the observations by inverted microscope, we found that the mycelium of JRF11 tightly contacted with the tomato root, and the mycelium was wrapped around the surface of the tomato root and colonized inside the root (Figure 3D). These results implied that JRF11 could successfully establish biotrophic interactions with tomato roots via attachment and invasion of mycelia to provide beneficial effects [35]. We also conducted sequencing of the ITS region and phylogenetic tree analysis and found that JRF11 was mostly close to the *Paraphaeosphaeria sardoa* strain (CBS 501.71) (Figure 3E). Furthermore, we sequenced the *18S rRNA* gene, another molecular marker, of JRF11, and phylogenetic tree analysis also revealed that JRF11 belonged to *Paraphaeosphaeria* spp., although the closest strain was *Paraphaeosphaeria viciae* (MFLU 15-1231) (Supplementary Figure S1). Taken together, JRF11 was finally identified as a *Paraphaosphaeria* strain.

### 3.5. Effects of Paraphaosphaeria Strain JRF11 on the Growth of Tomato Plants

**Figure 4:**
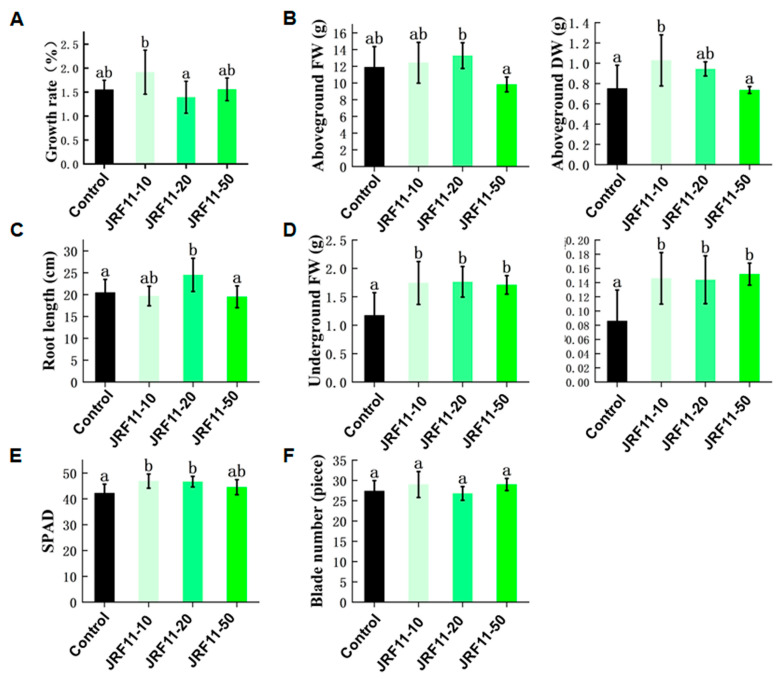
The growth indexes of tomato plants treated with *Paraphaosphaeria* strain JRF11 suspensions. Growth rate (**A**), aboveground FW and DW (**B**), root length (**C**), underground FW and DW (**D**), SPAD (**E**), and blade number (**F**) of tomato plants were recorded. FW represents fresh weight, and DW represents dry weight. JRF11-10, 10% stock solution. JRF11-20, 20% stock solution. JRF11-50, 50% stock solution. The data are the means ± standard deviation of five replicates. Different letters indicate significant differences between all treatments, according to Duncan’s multiple-range test (*p* < 0.05).

As JRF11 displayed a good effect in the plate test, in order to further verify the growth-promoting effect of JRF11, we selected different concentrations of JRF11 fermentation broth for the pot test. Results showed that compared with the control, JRF11-10 significantly increased the growth rate of the tomato plants (Figure 4A). Under the same conditions, the tomato plants treated with JRF11-10 and JRF11-20 showed an observable increase in overall growth and biomass of the plants, especially for the underground fresh/dry weight of JRF11-treated plants (Figure 4B,D). The root length of JRF11-20-treated plants was longer than that of the control (Figure 4C). And the biomass of the root increased significantly following all the treatments, JRF11-10, JRF11-20, and JRF11-50, compared to control plants (Figure 4D). In JRF11-inoculated tomato plants, both JRF11-10 and JRF11-20 significantly enhanced shoot and root biomass, while the largest increases in shoot and root biomass were observed in the plants inoculated with JRF11-10. In addition, JRF11-10 and JRF11-20 significantly increased the SPAD value of tomato plants (Figure 4E), but there was no significant difference in the number of leaves between JRF11-treated and control plants (Figure 4F). In summary, we found that JRF11-10 treatment significantly promoted the growth of tomato plants.

### 3.6. Effects of Paraphaosphaeria Strain JRF11 on Physiology of Tomato Plants

**Figure 5:**
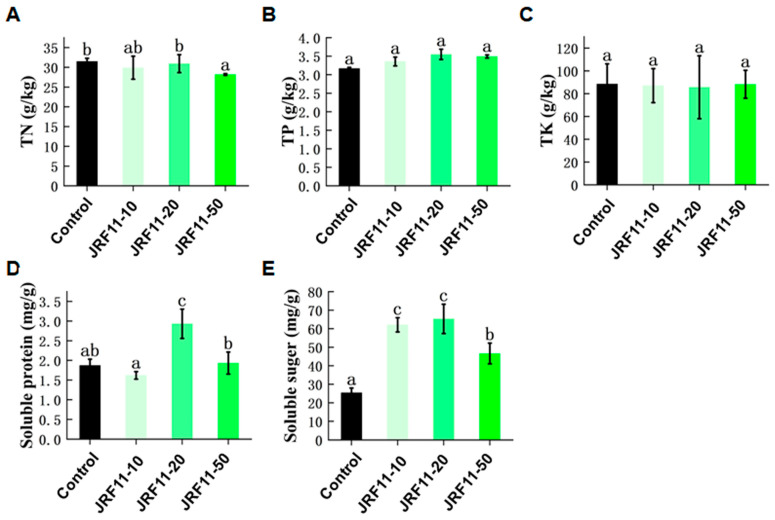
The physiology indexes of tomato plants treated with *Paraphaosphaeria* strain JRF11 suspensions. TN (**A**), TP (**B**), TK (**C**), soluble protein (**D**), and soluble sugar (**E**) of tomato plants were recorded. TN represents total nitrogen, TP represents total phosphorus, and TK represents total potassium. Values are the means ± standard deviation of four replications. According to Duncan’s multiple-range test (*p* < 0.05), different letters indicate significant differences between all treatments.

In order to investigate the effect of JRF11 on the physiology of inoculated plants, we measured the index of total nitrogen (TN), total phosphorus (TP), total potassium (TK), soluble protein, and soluble sugar. Results showed that the content of TN, TP, and TK in plants did not change significantly after JRF11 treatment, but the content of TP increased in each JRF11 treatment, including JRF11-10, JRF11-20, and JRF11-50 (Figure 5A–C). Soluble protein was an important osmoregulatory substance and nutrient in plants. Most soluble proteins were enzymes involved in various metabolisms, which can regulate the growth of plants and protect plants against stress [36]. Results showed that the content of soluble protein in JRF11-20-treated plants was significantly increased compared with the control (Figure 5D). Soluble sugar can provide energy and metabolic intermediates for the growth and development of plants. It can also form complex signal networks with plant hormones to regulate the growth and development of plants [37]. As shown in Figure 5E, the content of soluble sugar in all JRF11-treated plants was significantly increased. In particular, JRF11-10 and JRF11-20 treatments dramatically and significantly increased the soluble sugar content of tomato plants.

### 3.7. Transcriptome Analysis of Paraphaosphaeria sp. JRF11-Treated Plants

**Figure 6:**
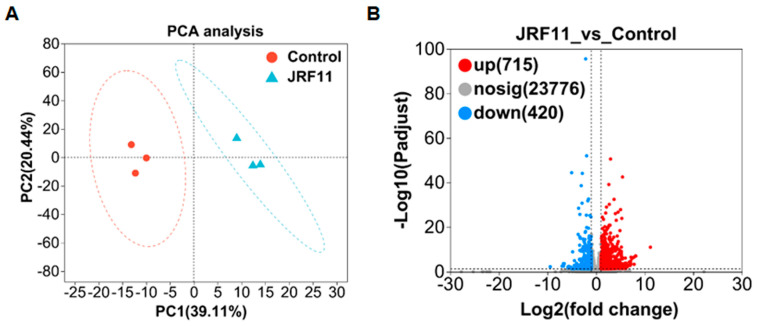
PCA analysis (**A**) and volcano plot (**B**) of DEGs between control and JRF11 samples.

In order to explain the growth-promoting effect of JRF11 on tomato plants, we did RNA sequencing to profile the changes in gene expression. A total of six samples were designed for transcriptome analysis, including three JRF11-treated samples and three control samples. PCA (principal component analysis) analysis showed that JRF11 and control samples were mainly located in the first and fourth quadrants and the second and third quadrants, respectively (Figure 6A). There was a significant difference between the two samples. PC1 and PC2 contributed 39.11% and 20.44%, respectively (Figure 6A). Second, differentially expressed genes (DEGs) were defined based on the criteria (|fold change, FC| ≥ 2 and Q-value ≤ 0.001). The number of DEGs was depicted as volcano plots. Compared with the control, JRF11 treatment resulted in 715 up-regulated and 420 down-regulated genes (Figure 6B). These DEGs included genes related to root development, secondary metabolite synthesis, etc. (Supplementary Table S1).

**Figure 7:**
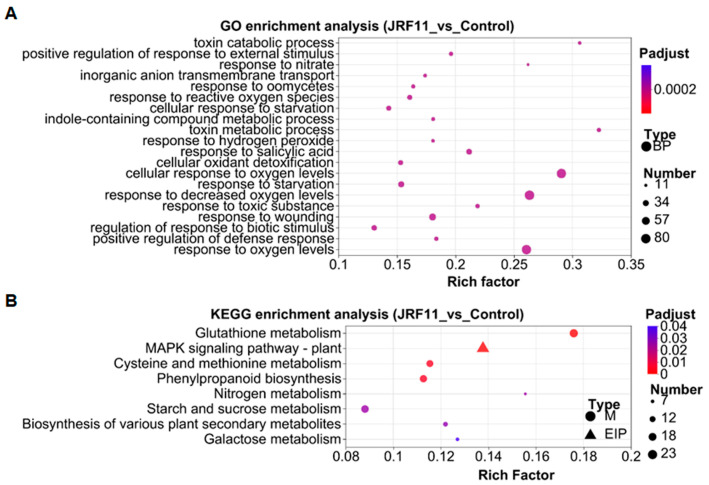
Gene Ontology (GO) enrichment analysis (**A**) and Kyoto Encyclopedia of Genes and Genomes (KEGG) enrichment analysis (**B**) of DEGs between Control and JRF11 groups. The X-axis represents the enrichment factor, and the Y-axis represents the pathway name. The depth of color represents the Q-value, and the size of the dot represents the number of DEGs.

In order to explore the related biological processes of these DEGs, we conducted gene ontology (GO) enrichment analysis based on the Fisher test. We observed that the three prominent enriched GO terms under JRF11 treatment were “cellular response to oxygen levels”, “response to decreased oxygen levels” and “response to oxygen levels” (Figure 7A). Oxygen has been reported to be involved in the regulation of plant growth, development, and differentiation [38]. This result suggested that JRF11 might promote plant growth through regulation of oxygen level-related genes in plants.

The Kyoto Encyclopedia of Genes and Genomes (KEGG) enrichment analysis was also performed to further understand the DEGs. Compared with the control, the major enriched pathways by JRF11 treatment were “glutathione metabolism”, “mitogen-activated protein kinase (MAPK) signaling pathway-plant”, “cysteine and methionine metabolism”, “phenylpropanoid biosynthesis”, and “starch and sucrose metabolism” (Figure 7B). Among them, plant MAPK signaling participates in plant growth and development, especially for primary root development and LR (lateral root) emergence [39]. Starch and sucrose metabolism also play pivotal roles in development; they can improve the growth of plants [40]. These results were consistent with the increase in root biomass and soluble sugar content in tomato plants with the JRF11 treatment.

## 4. Discussion

In this study, we have successfully isolated and identified 14 strains of endophytic fungi from *Carya illinoinensis*. These endophytic fungi were identified based on morphological characteristics and ITS sequencing. Among them, four strains (JRF2, JRF5, JRF7, and JRF9) might be harmful to plants like phytopathogens in early studies. To test whether these endophytic fungi can promote plant growth, we conducted plant–fungus interaction assays. Results showed that JRF1 and JRF11 had positive effects on plant growth, while JRF3, JRF6, JRF8, JRF10, and JRF14 had negative effects, and JRF4, JRF12, and JRF13 had little effect. In addition, JRF11 has an outstanding positive effect on plant growth and development. Many fungi have been shown to have growth-promoting effects on plants. For example, 128 fungal isolates, representing 31 genera and 37 species, were isolated from five plants on the west coast of Korea, and *Gibberella intermedia* fungal isolates significantly increased the growth of plants [41]. The endophytic fungus *P. citrinum*, isolated from the roots of *Ixeris repens*, promoted the growth of waito-c rice and *Atriplex gemelinii* seedlings [42]. In another study, the fungal endophyte *Acremonium* sp. Ld-03 was isolated from the bulbs of *L. davidii*. Application of its 40% culture dilution resulted in a significant increase in root and shoot length of *Allium tuberosum* [43]. Furthermore, Su. et al. isolated *Paraphaeosphaeria* sp. YXG-18 from the medicinal plant *Ginkgo biloba* and found that the interaction between endophytes and the host could induce the production of antifungal metabolites against pathogens for plant resistance [16]. Therefore, whether JRF11 can induce host resistance to pathogens will be tested in future studies.

In our study, we used JRF11 to inoculate tomato seedlings and found that the mycelium of JRF11 densely surrounds the root of tomato seedlings. These results indicated that the mycelium of JRF11 could successfully attach to or enter the roots of tomato seedlings, and JRF11 could colonize tomato seedlings. Similar studies have shown that some fungi can colonize plant roots. Yan et al. found that after *Piriformospora indica* became symbiotic with plants, its hyphae could be detected around the primary and lateral roots, and mycelia started to invade the root tissue [44]. Sujit Shah et al. found that the fungus *Coniochaeta* sp., which was isolated from the roots of *Dendrobium longicornu* Lindl, could successfully colonize and promote the growth of *Cymbidium aloifolium* [45]. Therefore, the colonization of the endophytic fungi JRF11 on plant roots contributes to its plant growth-promoting effects.

Based on the growth data of tomato plants, we found that the underground fresh weight/dry weight and root length of plants inoculated with JRF11 were significantly increased. These data indicate that JRF11 has a positive effect on plant root growth and development. JRF11 not only increased the number of lateral roots of the plant, making the root system more developed, but also increased the root length of the plant. Similar results were reported by Muhammad Aizaz, who found an increase in the plant growth characteristics of wheat with the inoculation of the fungi GREF2 and TQRF8. In particular, these isolated fungi significantly affected the root length of wheat [46]. In addition, we observed that the content of nitrogen, phosphorus, and potassium was not significantly changed in tomato plants inoculated with JRF11 fermentation solutions. While the content of soluble sugar was significantly increased in JRF11-inoculated plants, Li et al. found that the soluble sugar content of whole plants was higher under drought stress after *Suillus luteus* inoculation, indicating that *Suillus luteus* activated more accumulation of soluble sugar to respond to drought stress, which was conducive to improving the osmotic adjustment ability of plants [47]. Thus, we hypothesized that nitrogen, phosphorus, and potassium may be converted into other forms of nutrients to improve the growth of plants.

JRF11 treatment resulted in 715 up-regulated and 420 down-regulated genes. Based on GO enrichment analysis, we observed that “cellular response to oxygen levels”, “response to decreased oxygen levels”, and “response to oxygen levels” were the three prominent enriched GO terms under JRF11 treatment, suggesting JRF11 treatment led to the reprograming of oxygen level responses. Oxygen level is thought to play an important role in plant biology, which acts as an indispensable substrate for many biochemical reactions in plants, including energy metabolism (respiration). In addition, oxygen level is involved in many important signaling responses and is beneficial to plants by supporting cellular proliferation, physiological function, and viability [48,49]. Similarly, transcriptome analysis of *Piriformospora indica* indicated that the DEGs with the greatest changes were concentrated in oxidoreductase activity and ion transmembrane transporter activity [50]. KEGG enrichment analysis was also used to understand the DEGs after JRF11 treatment. The major enriched pathways by JRF11 treatment were “glutathione metabolism”, “MAPK signaling pathway-plant”, “cysteine and methionine metabolism”, “phenylpropanoid biosynthesis”, “starch and sucrose metabolism”, etc. The MAPK pathway is an important signaling event associated with every aspect of plant growth, development, and abiotic and biotic stress adaptation. Being a central metabolic pathway, it is a pivotal target for manipulation for crop improvement [51]. The phenylpropanoid biosynthesis pathway is usually activated under abiotic stress conditions, resulting in the accumulation of various phenolic compounds that have the potential to scavenge harmful reactive oxygen species [52]. Furthermore, starch and sucrose metabolism enhance plant resistance to external environmental stresses, with stored starch providing resources that enable plants to recover quickly after rehydration [53]. Through KEGG enrichment analysis, Mustafa Erayman et al. also observed that the most altered transcripts were associated with starch and sucrose metabolism and gluconeogenesis pathways [54]. Therefore, the above pathways may play an important role in plant growth and development, and these results are further consistent with our finding that JRF11 significantly increased root biomass and soluble sugar in tomato plants.

## Figures and Tables

**Table 1:** List of 14 endophytic fungi of *Carya illinoinensis*.

Name	Tissue	Closest Species	Identity	Accession Number	Effect	Description of Colonies
JRF1	Stem	*Aureobasidium melanogenum* CBS 105.22	98.14%	NR 159598.1	+	The hyphae are grayish green and flocculent. The colony margin is smooth and army green.
JRF2	Stem	*Botryosphaeria wangensis* CGMMCC 3.18744	100%	NR 159555.1	/	There are grayish green punctate secreta on the surface of the colony. The hyphae are white, but the center of the colony is grayish green with a radiation line.
JRF3	Stem	*Diaporthe alnea* CBS 146.46	97.52%	NR 147525.1	-	The hyphae are white. The colony with an annular line looks like a petal; it produces few aerial hyphae.
JRF4	Stem	*Diaporthe celeris*	98.86%	NR 158433.1	*	The hyphae look like white cotton and produce aerial hyphae. The colony looks like concentric circles; there is brown sediment in the center of the colony.
JRF5	Stem	*Diaporthe longicolla* ATCC 60325	97.91%	NR 144924.1	/	The hyphae look like white cotton; they produce aerial hyphae. The colony changes from white to yellow.
JRF6	Stem	*Diaporthe sackstonii* BRIP 54669b	98.85%	NR 147537.1	-	The hyphae look like white cotton. There are punctate secreta and few aerial hyphae on the surface of the colony. Its margin produces pigmentation.
JRF7	Stem	*Diaporthe sojae* CBS 139282	99.43%	NR 147542.1	/	The hyphae are white, and the distribution of aerial hyphae is tight. The colony with a line looks like a petal.
JRF8	Stem	*Diaporthe vaccinii* CBS 160.32	97.53%	NR 103701.1	-	The hyphae are white. The colony with an annular line looks like a petal; the distribution of aerial hyphae is tight, but its margin produces yellow pigmentation at the end.
JRF9	Root	*Fusarium foetens* CBS 110286	100%	NR 159865.1	/	The hyphae are white and villiform. The colony is reddish brown with little aerial hyphae.
JRF10	Root	*Mycoleptodiscus terrestris* CBS 231.53	99.48%	NR 145373.1	-	The colony changes from grayish green to atrovirens. A large number of punctate granules are distributed on the surface and inside the medium.
JRF11	Root	*Paraphaeosphaeria sardoa* CBS 501.71	97.81%	NR 145167.1	+	The hyphae are white and villiform. The colony looks like concentric circles, and the margin of the colony is smooth. The growth of this fungus is slow.
JRF12	Root	*Penicillium javanicum* CBS 341.48	92.16%	NR 111511.1	*	The hyphae produce a radiation line. The colony is yellow, and the punctate granule is distributed on the surface of the colony.
JRF13	Root	*Pseudthielavia terricola* CBS 603.97	99.22%	NR 165513.1	*	The hyphae are white. The colony with a line looks like a petal and the aerial hyphae look like white cotton.
JRF14	Root	*Talaromyces coprophilus* FMR 15199	99.09%	NR 172395.1	-	The hyphae are villiform. There are yellow punctations on the surface of the colony, and they distribute like concentric circles.

Notes: + represents positive effect, - represents negative effect, * represents no obvious effect, / represents untested.

**Table 2:** The growth rate and sporulation quantity of *Paraphaosphaeria* sp. JRF11 on different media.

Medium	Growth Rate (mm/d)	Sporulation Quantity
Potato Dextrose Agar (PDA)	5.50	II
Yeast Extract Peptone Dextrose Medium	5.42	I
Modified Melin-Norkrans Medium	4.40	I
Corn Meal Medium	5.00	III
Czapek Dox Agar Medium	4.69	I
Malt Extract Agar	5.00	II
Sabouraud Dextrose Agar Medium	4.13	I

Notes: I: <5/μL, II: 5–10/μL, III: >10/μL.

## Data Availability

Data are contained within the article and supplementary materials.

## Supplementary Materials

The following supporting information can be downloaded at: https://www.mdpi.com/article/10.3390/jof10020120/s1, Table S1: DEGs between the control and JRF11 groups. Figure S1: Phylogenetic tree based on *18S rRNA* gene sequences.
