# Supplementary figures and images for: Identification and Characterization of a Plant Endophytic Fungus Paraphaosphaeria sp. JRF11 and Its Growth-Promoting Effects
Source: J Fungi (Basel). 2024 Jan 31;10(2):120. doi: 10.3390/jof10020120 (PMC10890554; doi:10.3390/jof10020120)

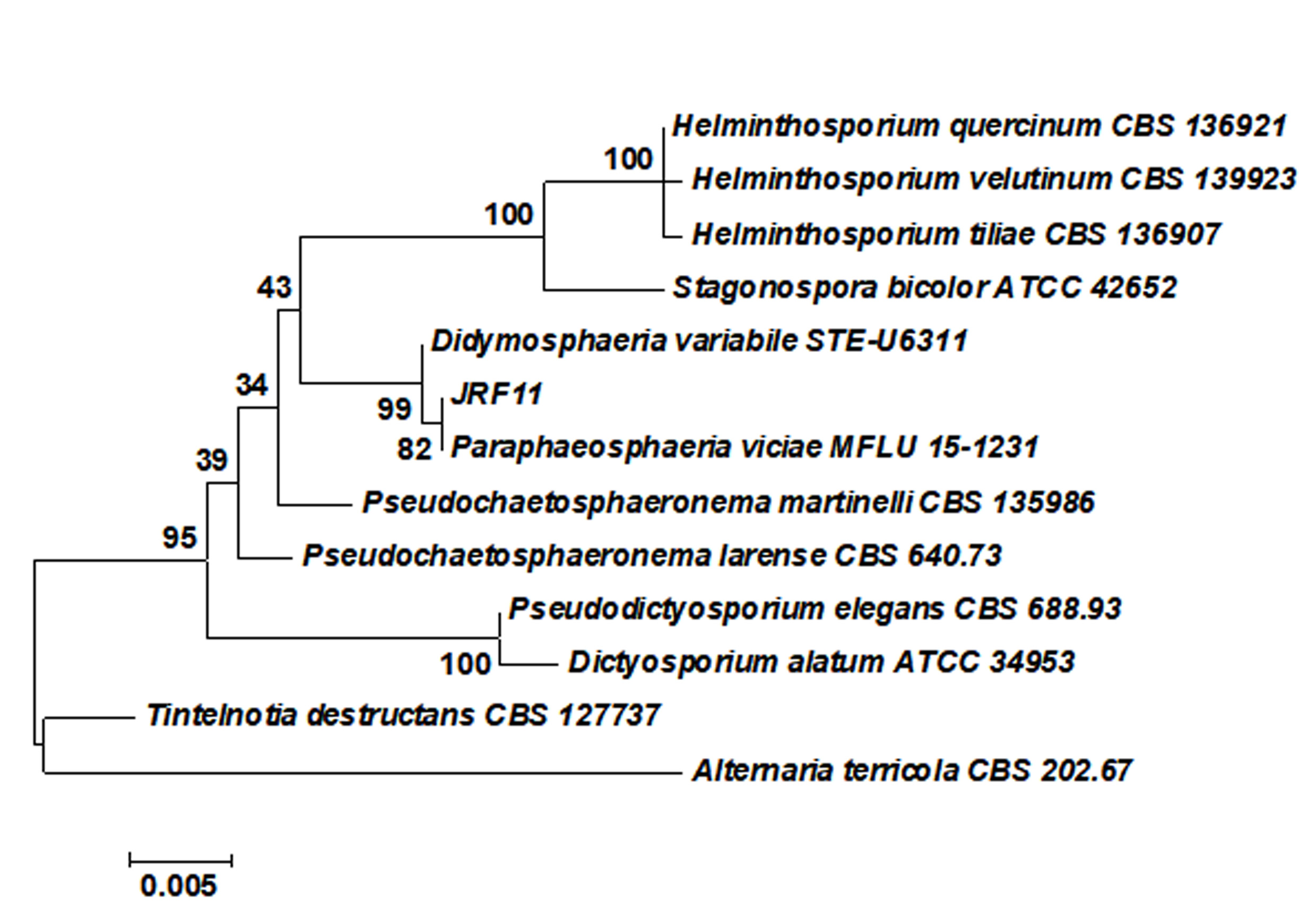

Supplement: Supplementary file 1 [file jof-10-00120-s001.zip › Figure S1 Phylogenetic tree based on 18S rRNA gene sequences.jpg]
